# Supplementary material for: Site-directed MT1-MMP trafficking and surface insertion regulate AChR clustering and remodeling at developing NMJs
Source: eLife. 2020 Mar 24;9:e54379. doi: 10.7554/eLife.54379 (PMC7093154; doi:10.7554/eLife.54379)
Supplement: Reporting standard 1. [file elife-54379-repstand1.docx]

| Figure | Comparison | | Test | Sample size | Number of independent experiments | *p*-value | Asterisks | post hoc tests | F, Dfn, Dfd |
| --- | --- | --- | --- | --- | --- | --- | --- | --- | --- |
| 1B | Top | PDL vs. ECL | One-way ANOVA | 300 muscle cells in each condition | 3 | 0.0465 | * | Dunnett's multiple comparisons test | 2.527, 4, 10 |
|  |  | PDL vs. Laminin |  |  |  | 0.2053 | n.s. |  |  |
|  |  | PDL vs. Collagen |  |  |  | 0.3634 | n.s. |  |  |
|  |  | PDL vs. Gelatin |  |  |  | 0.8377 | n.s. |  |  |
|  | Bottom | PDL vs. ECL | One-way ANOVA | 300 muscle cells in each condition | 3 | 0.0344 | * | Dunnett's multiple comparisons test | 9.792, 4, 10 |
|  |  | PDL vs. Laminin |  |  |  | 0.0089 | ** |  |  |
|  |  | PDL vs. Collagen |  |  |  | 0.0004 | *** |  |  |
|  |  | PDL vs. Gelatin |  |  |  | 0.0069 | ** |  |  |
| 1E |  | PDL vs. ECL | One-way ANOVA | 150 muscle cells in each condition | 3 | 0.0042 | ** | Dunnett's multiple comparisons test | 6.97, 4, 10 |
|  |  | PDL vs. Laminin |  |  |  | 0.0133 | * |  |  |
|  |  | PDL vs. Collagen |  |  |  | 0.0087 | ** |  |  |
|  |  | PDL vs. Gelatin |  |  |  | 0.0047 | ** |  |  |
| 2B |  |  | Pearson's correlation | 84 muscle cells | 3 | <0.0001 | - | / | / |
| 2F | 1 Day | Control vs. BB-94 | Two-way ANOVA | > 280 muscle cells in each condition | 3 | 0.8995 | n.s. | Dunnett’s multiple comparisons test | Time point: 207.1, 3, 6 Treatment: 275.5, 2, 4 Time point x Treatment Interaction: 50.67, 6, 12 |
|  |  | Control vs. BB-2516 |  |  |  | 0.5591 | n.s. |  |  |
|  | 2 Days | Control vs. BB-94 |  |  |  | <0.0001 | **** |  |  |
|  |  | Control vs. BB-2516 |  |  |  | <0.0001 | **** |  |  |
|  | 3 Days | Control vs. BB-94 |  |  |  | <0.0001 | **** |  |  |
|  |  | Control vs. BB-2516 |  |  |  | <0.0001 | **** |  |  |
|  | 4 Days | Control vs. BB-94 |  |  |  | <0.0001 | **** |  |  |
|  |  | Control vs. BB-2516 |  |  |  | <0.0001 | **** |  |  |
| 2G |  | Control vs. BB-94 | One-way ANOVA | 150 muscle cells in each condition | 3 | 0.4093 | n.s. | Dunnett’s multiple comparisons test | 0.8857, 2, 6 |
|  |  | Control vs. BB-2516 |  |  |  | 0.5183 | n.s. |  |  |
| 2H |  | Control vs. BB-94 | One-way ANOVA | 166 (Control), 154 (BB-94), 137 (BB-2516) muscle cells | 3 | 0.0005 | *** | Dunnett’s multiple comparisons test | 37.2, 2, 6 |
|  |  | Control vs. BB-2516 |  |  |  | 0.0007 | *** |  |  |
| 2J | 24 h | Control vs. BB-94 | Two-way ANOVA | 43 (Control) and 38 (BB-94) muscle cells | 3 | 0.9975 | n.s. | Sidak’s multiple comparisons test | Interaction: 14.48, 3, 237 Time point: 174.9, 3, 237 Treatment: 5.969, 1, 79 Subjects (matching): 8.392, 79, 237 |
|  | 48 h |  |  |  |  | 0.9981 | n.s. |  |  |
|  | 72 h |  |  |  |  | 0.0146 | * |  |  |
|  | 96 h |  |  |  |  | <0.0001 | **** |  |  |
| 3C | Top | MT1-mCherry vs. Control | One-way ANOVA | 163 (Control), 123 (MT1-mCherry), 119 (MT1-mCherry + BB-94), and 62 (MT1-mCherry + BB-2516) muscle cells | 4 | 0.0019 | ** | Dunnett's multiple comparisons test | 8.412, 3, 10 |
|  |  | MT1-mCherry vs. MT1-mCherry + BB-94 |  |  |  | 0.1304 | n.s. |  |  |
|  |  | MT1-mCherry vs. MT1-mCherry + BB-2516 |  |  |  | 0.017 | * |  |  |
|  | Bottom | MT1-mCherry vs. Control | One-way ANOVA |  |  | 0.0016 | ** | Dunnett's multiple comparisons test | 11.09, 3, 10 |
|  |  | MT1-mCherry vs. MT1-mCherry + BB-94 |  |  |  | 0.0022 | ** |  |  |
|  |  | MT1-mCherry vs. MT1-mCherry + BB-2516 |  |  |  | 0.0073 | ** |  |  |
| 3D | AChR | Control vs. MT1-mCherry | One-way ANOVA | 14 (Control), 19 (MT1-mCherry), 21 (MT1-mCherry + BB-94), and 16 (MT1-mCherry + BB-2516) muscle cells | 3 | 0.0156 | * | Turkey’s multiple comparisons test | 6.809, 3, 8 |
|  |  | Control vs. MT1-mCherry + BB-94 |  |  |  | 0.4956 | n.s. |  |  |
|  |  | Control vs. MT1-mCherry + BB-2516 |  |  |  | 0.9911 | n.s. |  |  |
|  |  | MT1-mCherry vs. MT1-mCherry + BB-94 |  |  |  | 0.1203 | n.s. |  |  |
|  |  | MT1-mCherry vs. MT1-mCherry + BB-2516 |  |  |  | 0.023 | * |  |  |
|  |  | MT1-mCherry + BB-94 vs. MT1-mCherry + BB-2516 |  |  |  | 0.653 | n.s. |  |  |
|  | Gelatin | Control vs. MT1-mCherry | One-way ANOVA | 30 (Control), 39 (MT1-mCherry), 33 (MT1-mCherry + BB-94), and 31 (MT1-mCherry + BB-2516) muscle cells | 3 | <0.0001 | **** | Turkey’s multiple comparisons test | 105.2, 3, 8 |
|  |  | Control vs. MT1-mCherry + BB-94 |  |  |  | 0.9782 | n.s. |  |  |
|  |  | Control vs. MT1-mCherry + BB-2516 |  |  |  | 0.9252 | n.s. |  |  |
|  |  | MT1-mCherry vs. MT1-mCherry + BB-94 |  |  |  | <0.0001 | **** |  |  |
|  |  | MT1-mCherry vs. MT1-mCherry + BB-2516 |  |  |  | <0.0001 | **** |  |  |
|  |  | MT1-mCherry + BB-94 vs. MT1-mCherry + BB-2516 |  |  |  | 0.9959 | n.s. |  |  |
| 4B | AChR | Control vs. CLASP-MO | Unpaired t-test | 16 (Control), and 13 (CLASP-MO) cells | 3 | 0.2329 | n.s. | / | / |
|  | Non-AChR | Control vs. CLASP-MO |  |  |  | 0.9591 | n.s. |  |  |
|  | Control | AChR vs. non-AChR |  |  |  | 0.0021 | ** |  |  |
|  | CLASP-MO | AChR vs. non-AChR |  |  |  | 0.0117 | * |  |  |
| 4C | AChR | Control vs. CLASP-MO | Unpaired t-test | 16 (Control), and 13 (CLASP-MO) cells | 3 | 0.0144 | * | / | / |
|  | Non-AChR | Control vs. CLASP-MO |  |  |  | 0.7992 | n.s. |  |  |
|  | Control | AChR vs. non-AChR |  |  |  | 0.0002 | *** |  |  |
|  | CLASP-MO | AChR vs. non-AChR |  |  |  | 0.0686 | n.s. |  |  |
| 4H |  | Control vs. CLASP-MO | Unpaired t-test | 18 (Control) and 14 (CLASP-MO) cells | 4 | 0.0341 | * | / | / |
| 6B |  | Control vs. Low MT1-pHluroin level | One-way ANOVA | 150 (Control), 32 (Low MT1-pHluorin level), and 15 (High MT1-pHluorin level) muscle cells | 3 | 0.0646 | n.s. | Bonferroni’s multiple comparisons test | 17.1, 2, 6 |
|  |  | Control vs. High MT1-pHluroin level |  |  |  | 0.0022 | ** |  |  |
| 6E |  |  | Pearson's correlation | 13 MT1-pHluorin expressing muscle cells | 3 | 0.0056 | - | / | / |
| 6H |  | Control vs. CLASP-MO | Unpaired t-test | 10 (Control) and 10 (CLASP-MO) muscle cells | 3 | 0.0004 | *** | / | / |
| 7B | AChR | Control vs. BB-94 | One-way ANOVA | 30 (Control), 32 (BB-94), and 38 (BB-2516) nerve-muscle pairs | 4 | 0.0002 | *** | Dunnett's multiple comparisons test | 30.34, 2, 9 |
|  |  | Control vs. BB-2516 |  |  |  | 0.0002 | *** |  |  |
|  | Gelatin | Control vs. BB-94 | One-way ANOVA | 30 (Control), 32 (BB-94), and 38 (BB-2516) nerve-muscle pairs | 4 | 0.0028 | ** | Dunnett's multiple comparisons test | 13.32, 2, 9 |
|  |  | Control vs. BB-2516 |  |  |  | 0.003 | ** |  |  |
| 7G |  | Control vs. BB-94 | One-way ANOVA | 30 (Control), 27 (BB-94), and 22 (BB-2516) muscle cells | 3 | 0.0101 | * | Dunnett's multiple comparisons test | 9.547, 2, 6 |
|  |  | Control vs. BB-2516 |  |  |  | 0.0354 | * |  |  |
| 8B |  | WT vs. Control MO | One-way ANOVA | 127 (WT), 120 (Control MO), 146 (MT1-MO), or 34 (MT1-mCherry + MT1-MO) nerve-muscle pairs | 3 | 0.8084 | n.s. | Sidak’s multiple comparisons test | 19.53, 3, 8 |
|  |  | WT vs. MT1-MO |  |  |  | 0.0007 | *** |  |  |
|  |  | WT vs. MT1-mCherry + MT1-MO |  |  |  | 0.906 | n.s. |  |  |
|  |  | Control MO vs. MT1-MO |  |  |  | 0.0029 | ** |  |  |
|  |  | Control MO vs. MT1-mCherry + MT1-MO |  |  |  | >0.9999 | n.s. |  |  |
|  |  | MT1-MO vs. MT1-mCherry + MT1-MO |  |  |  | 0.0022 | ** |  |  |
| 8C |  | WT vs. Control MO | One-way ANOVA | 29 (WT), 30 (Control MO), 32 (MT1-MO), or 23 (MT1-mCherry + MT1-MO) nerve-muscle pairs | 4 | 0.6984 | n.s. | Sidak’s multiple comparisons test | 7.597, 3, 8 |
|  |  | WT vs. MT1-MO |  |  |  | 0.0058 | ** |  |  |
|  |  | WT vs. MT1-mCherry + MT1-MO |  |  |  | 0.4882 | n.s. |  |  |
|  |  | Control MO vs. MT1-MO |  |  |  | 0.0245 | * |  |  |
|  |  | Control MO vs. MT1-mCherry + MT1-MO |  |  |  | 0.9803 | n.s. |  |  |
|  |  | MT1-MO vs. MT1-mCherry + MT1-MO |  |  |  | 0.0415 | * |  |  |
| 8D |  | WT vs. Control MO | One-way ANOVA | 24 (WT), 23 (Control MO), 25 (MT1-MO), or 19 (MT1-mCherry + MT1-MO) nerve-muscle pairs | 3 | 0.9989 | n.s. | Sidak’s multiple comparisons test | 10.09, 3, 8 |
|  |  | WT vs. MT1-MO |  |  |  | 0.0129 | * |  |  |
|  |  | WT vs. MT1-mCherry + MT1-MO |  |  |  | 0.9982 | n.s. |  |  |
|  |  | Control MO vs. MT1-MO |  |  |  | 0.0074 | ** |  |  |
|  |  | Control MO vs. MT1-mCherry + MT1-MO |  |  |  | 0.9504 | n.s. |  |  |
|  |  | MT1-MO vs. MT1-mCherry + MT1-MO |  |  |  | 0.0243 | * |  |  |
| 9C | From aneural AChR clusters + diffuse AChRs | WT vs. Control MO | Two-way ANOVA | From aneural AChR clusters + diffuse AChRs (without photobleaching): 15 (WT), 9 (Control MO), and 9 (MT1-MMP MO) nerve-muscle pairs; From diffuse AChRs (with photobleaching): 23 (WT), 13 (Control MO), and 8 (MT1-MMP MO) nerve-muscle pairs | 3 | >0.9999 | n.s. | Sidak’s multiple comparisons test | AChR source: 23.3, 1, 2 Treatment: 10.47, 2, 4 AChR source x Treatment Interaction: 9.249, 2, 4 |
|  |  | WT vs. MT1-MMP MO |  |  |  | 0.0255 | * |  |  |
|  |  | Control MO vs. MT1-MMP MO |  |  |  | 0.0255 | * |  |  |
|  | From diffuse AChRs | WT vs. Control MO | Two-way ANOVA |  | 3 | 0.9996 | n.s. | Sidak’s multiple comparisons test |  |
|  |  | WT vs. MT1-MMP MO |  |  |  | 0.9994 | n.s. |  |  |
|  |  | Control MO vs. MT1-MMP MO |  |  |  | 0.9821 | n.s. |  |  |
|  | WT | From aneural AChR clusters + diffuse AChRs vs. From diffuse AChRs | Two-way ANOVA |  | 3 | 0.0071 | ** | Sidak’s multiple comparisons test | Interaction: 7.342, 2, 6 AChR source: 32.9, 1, 6 Treatment: 12.9, 2, 6 Subjects (matching): 0.8463, 6, 6 |
|  | Control MO |  |  |  |  | 0.0098 | ** |  |  |
|  | MT1-MMP MO |  |  |  |  | 0.9971 | n.s. |  |  |
| 10D | E13.5 | WT vs. MT1-MMP^-/-^ | Two-way ANOVA | 4 (E13.5, WT) and 5 (E13.5, MT1-MMP^-/-^), 5 (E18.5, WT), and 3 (E18.5, MT1-MMP^-/-^) embryos | 3 | 0.0033 | ** | Sidak’s multiple comparisons test | Interaction: 8.001, 1, 13 Stage: 367.8, 1, 13 Genotype: 6.382, 1, 13 |
|  | E18.5 | WT vs. MT1-MMP^-/-^ |  |  |  | 0.9746 | n.s. |  |  |
| 10E | E13.5 | WT vs. MT1-MMP^-/-^ | Two-way ANOVA |  |  | 0.065 | n.s. | Sidak’s multiple comparisons test | Interaction: 4.466, 1, 13 Stage: 47.66, 1, 13 Genotype: 28.51, 1, 13 |
|  | E18.5 | WT vs. MT1-MMP^-/-^ |  |  |  | 0.0004 | *** |  |  |
| 10F | E13.5 | WT vs. MT1-MMP^-/-^ | Unpaired t-test |  |  | 0.3167 | n.s. | / | / |
|  | E18.5 | WT vs. MT1-MMP^-/-^ |  |  |  | 0.0002 | *** |  |  |
| 10G | E13.5 | WT vs. MT1-MMP^-/-^ | Unpaired t-test |  |  | 0.0175 | * | / | / |
|  | E18.5 | WT vs. MT1-MMP^-/-^ |  |  |  | 0.0011 | ** |  |  |
| 7-S1A | 0 h | Control vs. BB-94 | Two-way ANOVA | 18 (Control), 22 (BB-94), 20 (BB-2516) muscle cells | 3 | >0.9999 | n.s. | Dunnett's multiple comparisons test | Interaction: 3.599, 6, 171 Timepoint: 336.8, 3, 171 Treatment: 6.178, 2, 57 Subjects (matching): 3.938, 57, 171 |
|  |  | Control vs. BB-2516 |  |  |  | >0.9999 | n.s. |  |  |
|  | 4 h | Control vs. BB-94 |  |  |  | 0.044 | * |  |  |
|  |  | Control vs. BB-2516 |  |  |  | 0.092 | n.s. |  |  |
|  | 8 h | Control vs. BB-94 |  |  |  | 0.0058 | ** |  |  |
|  |  | Control vs. BB-2516 |  |  |  | 0.0106 | * |  |  |
|  | 24 h | Control vs. BB-94 |  |  |  | <0.0001 | **** |  |  |
|  |  | Control vs. BB-2516 |  |  |  | 0.0004 | *** |  |  |
| 7-S1C | 8 h | Control vs. BB-94 | Two-way ANOVA | 18 (Control), 22 (BB-94), 20 (BB-2516) muscle cells | 3 | 0.6465 | n.s. | Turkey’s multiple comparisons test | Time point: 3.804, 1, 2 Treatment: 0.1945, 2, 4 Time point x Treatment Interaction: 0.4187, 2, 4 |
|  |  | Control vs. BB-2516 |  |  |  | 0.8683 | n.s. |  |  |
|  |  | BB-94 vs. BB-2516 |  |  |  | 0.9071 | n.s. |  |  |
|  | 24 h | Control vs. BB-94 |  |  |  | 0.9812 | n.s. |  |  |
|  |  | Control vs. BB-2516 |  |  |  | 0.8743 | n.s. |  |  |
|  |  | BB-94 vs. BB-2516 |  |  |  | 0.7826 | n.s. |  |  |
| 7-S2B | 4 h | Control vs. Agrin | Unpaired t-test | 67 (Control, 4 h), 92 (Agrin, 4 h), 118 (Control, 8 h), and 93 (Agrin, 8 h) muscle cells | 3 | 0.0076 | ** | / | / |
|  | 8 h | Control vs. Agrin | Unpaired t-test |  | 3 | 0.0005 | *** | / | / |

­­
